# *Xanthium strumarium* L. Exhibits Potent Antiplatelet and Antithrombotic Effects by Modulating MAPK and PI3K/AKT Signaling Pathways and Inhibiting Ferric Chloride-Induced Thrombosis

**DOI:** 10.3390/biomedicines13122924

**Published:** 2025-11-28

**Authors:** Abdul Wahab Akram, Ga Hee Lee, Su-Min Baek, Jinsu Kang, Yoonhoi Koo, Yein Oh, Min-Soo Seo, Evelyn Saba, Dong-Ha Lee, Man Hee Rhee

**Affiliations:** 1 Department of Veterinary Medicine, College of Veterinary Medicine, Kyungpook National University, Daegu 41566, Republic of Korea; ab.wahab26@knu.ac.kr (A.W.A.);; 2 Department of Biomedical Laboratory Science, Molecular Diagnostics Research Institute, Namseoul University, Cheonan 31020, Republic of Korea; 3 Department of Veterinary Biomedical Sciences, Faculty of Veterinary and Animal Sciences, Pir-Mehr Ali Shah Arid Agriculture University, Rawalpindi 46000, Pakistan; 4 Institute for Veterinary Biomedical Science, College of Veterinary Medicine, Kyungpook National University, Daegu 41566, Republic of Korea

**Keywords:** *Xanthium strumarium* L., antiplatelet effects, anti-thrombotic effects, platelet hyperactivity, cardiovascular diseases

## Abstract

**Background:** Cardiovascular diseases, driven by platelet hyperactivation and thrombosis, remain the leading global cause of death. Excessive platelet activation contributes to atherosclerosis and thrombo-inflammatory disorders, underscoring the urgent need for safer and more effective antiplatelet agents. **Objectives:** *Xanthium strumarium* L. (*X. strumarium*) has been reported to exhibit a wide range of pharmacological effects, including anti-inflammatory and antioxidant activities. However, its antiplatelet and antithrombotic effects remain unexplored. Therefore, the present study aimed to comprehensively evaluate the antiplatelet and antithrombotic effects of *X. strumarium* through integrated in vitro and in vivo experiments. **Methods:** The principal bioactive compounds present in the *X. strumarium* extract were identified through GC–MS analysis. In vitro antiplatelet effects were evaluated via light transmission aggregometry, scanning electron microscopy (SEM), ATP and calcium mobilization assays, αIIbβ3 binding assay, clot retraction assay, and Western blotting. In vivo ferric chloride-induced (FeCl_3_) murine thrombus model was established to evaluate thrombogenesis. **Results:** Our results demonstrated that *X. strumarium* at 25, 50, or 100 μg/mL significantly inhibited collagen, ADP, U46619, and thrombin-induced platelet aggregation. SEM revealed that *X. strumarium* pretreatment markedly preserved the resting platelet morphology and inhibited collagen-induced activation and shape changes. Further, the granule secretion, integrin-αIIbβ3 signaling, and the MAPK and PI3K/Akt pathways were also concentration-dependently inhibited. The in vivo blood flow rate and mice survival were improved, and H&E staining further revealed a concentration-dependent prevention of arterial occlusion following *X. strumarium* treatment. **Conclusions:** Collectively, *X. strumarium* demonstrated potent antiplatelet and antithrombotic effects, improving blood flow and survival while preventing arterial occlusion.

## 1. Introduction

Cardiovascular diseases (CVD) represent a significant global health challenge, encompassing conditions that impair the heart and blood vessels [1]. CVD are the leading cause of death worldwide, with approximately 17.9 million deaths annually [2]. These conditions often result from fatty deposits that block blood flow, leading to complications such as coronary heart disease (CHD), stroke, myocardial infarction, and hypertension [3]. Platelet hyperactivity plays a crucial role in CVD pathophysiology by forming clots and causing vascular stenosis, which can lead to ischemic strokes [4,5]. Changes in blood flow dynamics cause arterial thrombus development and platelet activation at the sites of atherosclerotic plaque destruction [6]. Platelet adhesion is initiated through the interaction between the glycoprotein (GP) Ib–V–IX complex on platelets and von Willebrand factor (VWF) bound to subendothelial collagen, which subsequently promotes GPVI receptor engagement and triggers platelet activation [7]. This process generates thrombin and other mediators, which sustain platelet activation and coagulation. Thrombin also converts fibrinogen to fibrin, forming a network that covers and determines the microelasticity of thrombi [8,9]. Upon vascular injury, the exposure of the subendothelial matrix recruits platelets to form a hemostatic plug, with collagen binding to the GPVI receptor, initiating platelet activation [10]. This process includes shape change, granule secretion of ADP and thromboxane A2 (TXA2), and activation of integrin αIIbβ3, resulting in platelet activation and aggregation [10]. The generation of thrombin further stabilizes the platelet plug via protease-activated receptors (PARs). Critical signaling pathways, such as those involving MAPKs and the PI3K/Akt pathway, are activated by various agonists, enhancing platelet activation and aggregation [11]. However, antithrombotic drugs target specific receptors, such as GPVI for collagen, P2Y12 for ADP, U46619 for thromboxane A2 (TXA2), and PAR for thrombin to inhibit platelet function [12].

Although synthetic drugs like clopidogrel and aspirin (ASA) are used to treat CVD, they are associated with significant side effects, such as aplastic anemia, stomach ulcers, and drug resistance [13]. Ethnomedical approaches, which use natural herbal components and diets like the Mediterranean diet, offer promising alternatives for managing CVD. These natural strategies can help to modulate platelet activity and reduce thrombosis risk, potentially providing safer and more effective options for preventing and treating cardiovascular conditions when compared to synthetic drugs. Throughout history, plants have served as primary therapeutic resources for human ailments [14,15,16]. In modern times, the application of phytochemistry and pharmaceutical methods has facilitated the exploration of the mechanisms and chemical constituents of plants used in traditional medicine [17,18,19]. Numerous compounds derived from plants demonstrate biological activities capable of mitigating CVD [20,21,22]. Notably, flavonoids and carotenoids offer a diverse array of cardiovascular benefits [23,24].

*Xanthium* species are recognized for their diverse array of bioactive compounds, which contribute to their extensive pharmacological activities [25]. *Xanthium strumarium* L. (*X. strumarium*) has a long-standing history of medicinal use in China, Korea, Japan, Pakistan, and others [26]. In traditional Chinese medicine, the fruit of *X. strumarium*, known as Cang-Er-Zi, has been used for centuries to address conditions such as rhinitis, headaches, and rheumatic arthralgia [26]. The fruit is typically processed to enhance its efficacy and reduce toxicity. The therapeutic uses are also documented in Korea and Japan for similar indications, including anti-inflammatory and analgesic applications [27]. In Pakistan, *X. strumarium* is similarly valued for its therapeutic benefits [28]. In India, where it is referred to as Chotagokhru or Chotadhatura, the plant is used to treat leukoderma, insect bites, epilepsy, and biliousness [27]. Native American tribes have historically used *X. strumarium* to alleviate digestive issues [29], and in Bangladesh, it is applied to manage urinary disorders, ear infections, diabetes, and gastric disorders [30]. *X. strumarium* has been reported for its anti-inflammatory, antibacterial, antifungal, and antimalarial effects [27,31], and its high oil content suggests it has potential as a biodiesel feedstock [32]. M. Hassan et al. in 2022 reported that *X. strumarium* extract (from Al-Hada region near the Taif Governorate, Mecca Province, Saudi Arabia) exhibits antifungal activity against major pomegranate pathogens, with inhibition rates comparable to Nystatin [33]. Amin et al. in 2025 have reported the pharmacokinetic characteristics of several major compounds in *X. strumarium* (from Khyber Pakhtunkhwa, Pakistan) using in silico ADMET models, and the key constituents demonstrated favorable absorption, metabolism, and excretion [34]. Further, *X. strumarium* extract (from Goesan-gun, South Korea) inhibited melanogenesis by downregulating MITF and tyrosinase expression through GSK3β inactivation [35]. To the best of our knowledge, Korean *X. strumarium* has not previously been evaluated for its antiplatelet effects, nor systematically screened in the context of platelet function. Therefore, we hypothesized that *X. strumarium* inhibits platelet activation and aggregation by modulating key signaling pathways, including MAPK and PI3K/Akt, as addressed in this study.

## 2. Materials and Methods

### 2.1. Reagents

Collagen, ADP, and thrombin were obtained from Chrono-Log Co. (Havertown, PA 19083, USA). Thrombin from human plasma was purchased along with glutaraldehyde from Sigma-Aldrich (St. Louis, MO, USA). ATP assay kits were obtained from Cayman Chemical (Ann Arbor, MI, USA). PFA, Fura 2-AM, and Alexa Fluor 488-conjugated fibrinogen were obtained from Invitrogen (Eugene, OR, USA). Avertin was prepared as the anesthesia by dissolving 2,2,2-tribromoethanol (Sigma-Aldrich, catalog no. T48402-5G) in 2-methyl-2-butanol (Sigma-Aldrich, catalog no. 152463). Ferric chloride (FeCl_3_) was purchased from Sigma-Aldrich (Catalog no. 157740-100G). All Western blot antibodies were purchased from Cell Signaling Technology (Danvers, MA, USA).

### 2.2. Plant Material

The powdered form of the whole plant (mainly leaves and stems) was purchased from the Natural Product Central Bank (purchase no. KPM023-025, bar code no. PB4798.5) and kept at −20 °C until extraction.

### 2.3. Extraction of X. strumarium

*X. strumarium* extract was procured as published recently [36,37]. In brief, *X. strumarium* was extracted with 70% ethanol at 1:20 (*w*/*v*) at 80 °C for 2 h, filtered through filter paper (Whatman™ No. 4), evaporated using a rotary evaporator (R-100, BÜCHI Labortechnik, Flawil, Switzerland), and finally stored overnight in a freezer (−70 °C). The powdered *X. strumarium* was obtained after freeze-drying the extracted solvent for 3–4 days at −55 °C. Dimethyl sulfoxide (DMSO) was used to dissolve ethanol extracts at specific concentrations for subsequent sample evaluation.

### 2.4. GC-MS Analysis

GC-MS analysis was performed as reported previously [31], and the mass spectrometry data were collected using both scan and electron ionization modes to analyze the compounds present in the *X. strumarium* extract (Supplementary Table S1) [31].

### 2.5. Experimental Animals

Seven-week-old male Sprague-Dawley rats weighing 240–260 g were used for the in vitro platelet study, and male ICR mice weighing 30–40 g were used for the in vivo FeCl_3_-induced thrombus model as reported previously [38]. The sample size and power calculation used in this study were determined based on a previously published study by Irfan et al. [39]. Animals were acclimated to an environment control room maintained at approximately 23 ± 2 °C and 50 ± 10% humidity, with a 12 h light/dark cycle. All animal experiments were conducted according to accepted guidelines and received approval from the Animal Care Committee of the College of Veterinary Medicine, Kyungpook National University, Daegu, Republic of Korea (Permit no. KNU-2022-0083).

Human platelet-rich plasma (PRP) was obtained from the Blood Center of the Korean Red Cross, Suwon, Korea. The study protocol received clearance from the Institutional Review Board (IRB) under the Bioethics Review Committee of Namseoul University (1041479-BR-202208-005).

### 2.6. Light Transmission Aggregometry and Scanning Electron Microscopy (SEM)

Sprague-Dawley rat blood was collected via heart puncture using a syringe filled with acid-citrate-dextrose (ACD) to obtain purified platelets [20]. Washed platelets were effectively separated from the whole blood by centrifugation at 170× *g* for 7 min, followed by centrifugation at 350× *g* for 10 min. The isolated platelets were maintained at 3 × 10^8^ cells/mL for platelet aggregometry analysis. The platelets were incubated for 1 min with *X. strumarium* (25, 50, or 100 μg/mL) or with DMSO over an aggregometer in the presence of 1 mM CaCl_2_, and platelet aggregation was initiated by adding agonists (collagen at 2.5 μg/mL, ADP at 10 μM, or thrombin at 0.1 U/mL) for 5 min. This method allowed for the precise quantification of the platelet response under different experimental conditions, providing valuable insights into the effects of *X. strumarium* on platelet function.

The human PRP was spun in a centrifuge at a force of 1610× *g* for 8 min, followed by two washes with a buffer at pH 6.5, as previously outlined [40]. The resuspended pellets were prepared using a suspension buffer at pH 6.9. All processes were carried out at ambient temperature, and platelet suspensions were adjusted to reach a cell concentration of 10^8^ cells per milliliter. To assess the aggregation of platelets, human platelet suspensions (10^8^ cells/mL) were incubated with 1 mM of CaCl_2_ at 37 °C for 3 min, either with or without *X. strumarium* (25, 50, or 100 μg/mL) or with DMSO (negative control). Afterward, agonists (collagen 2.5 μg/mL, U46619 at 0.5 μM), or thrombin at 0.1 U/mL were added to stimulate the platelets. Platelet aggregation was monitored for 5 min under constant agitation.

For SEM, washed platelets were incubated with *X. strumarium* and agonists for 5 min. Platelet fixation was achieved using paraformaldehyde and osmium tetroxide (0.5% each), and then the platelets were dehydrated using increasing concentrations of ethanol from 50% to 100%. Subsequently, the platelets were freeze-dried at −55 °C. Ultrastructure images depicting platelet shape changes were obtained using a field emission electron microscope (SU8220, Hitachi, Tokyo, Japan).

### 2.7. Cytotoxicity Measurement

Platelets (2.5 × 10^8^/mL) were incubated with *X. strumarium* for 1 h, centrifuged at 12,000× *g*, and the supernatant was analyzed for LDH activity using an ELISA reader (TECAN, Salzburg, Austria).

### 2.8. ATP Release Assay, [Ca^2+^]_i_ Mobilization Assay, and Fibrinogen Binding Assay

This experiment involved preincubating platelets with the plant extract and then stimulating them with collagen to induce activation. Following this, ATP release assay, [Ca^2+^]_i_ mobilization assay, and fibrinogen binding assay were performed, as described previously [20]. ATP secretion was measured in the obtained supernatant using an ATP assay kit to assess platelet function. Additionally, [Ca^2+^]_i_ was analyzed using Fura-2/AM-loaded platelets using the following formula: 224 nM (*F* − *F*_min_)/(*F*_max_ − *F*). For the fibrinogen binding assay, the platelets were stained with an anti-fibrinogen antibody, and platelet activation levels were further evaluated through flow cytometry following pretreatment with collagen in the presence or absence of the plant extract.

### 2.9. Clot Retraction

Platelet-rich plasma (PRP, 250 μL) was incubated for 2 min with the vehicle, *X. strumarium*, or Y-27632 (a Rho kinase [ROCK] inhibitor). A final volume of 1 mL was obtained by adding red blood cells (5 μL) and Tyrode’s buffer to the mixture. Following this, a 1 U/mL injection of thrombin was administered, and the clot retraction was evaluated at room temperature. The thrombin clots were weighed to compare the clot retraction efficacy of different groups.

### 2.10. Fibronectin Adhesion Assay and Platelet Spreading on Immobilized Fibrinogen

Fibronectin adhesion assay and platelet spreading were evaluated as previously described [39]. With minor modifications in platelet spreading on immobilized fibrinogen, glass coverslips (22 × 22 mm) were coated with fibrinogen (100 µg/mL) for 2 h at 37 °C, rinsed with PBS, and blocked with 1% BSA for 1 h. Washed rat platelets (1.2 × 10^8^/mL) were preincubated with vehicle, *X. strumarium* extract (50 or 100 µg/mL), or GR155053 for 10 min at 37 °C. Platelets were then allowed to adhere and spread on the coated coverslips for 60 min. Following incubation, cells were fixed with 2% paraformaldehyde for 15 min and permeabilized with 0.2% Triton X-100 for 5 min. F-actin was stained with phalloidin (1:300) for 30 min in the dark. Coverslips were washed, mounted, and imaged using a Nikon(Tokyo, Japan) A1-R confocal microscope.

### 2.11. Western Blotting

After preincubating with *X. strumarium* and stimulation with collagen, lysis buffer was added to start lysis, and the resulting protein concentrations were quantified, and whole platelet proteins were isolated for subsequent analysis. These proteins were separated using SDS-PAGE and transferred to poly vinylidene fluoride (PVDF) membranes. The membranes were then blocked, and primary antibodies were applied overnight, followed by treatment with secondary antibodies for 3 h and washed three times for visualization using enhanced chemiluminescence. This comprehensive process allowed for the examination of changes in protein expression induced by *X. strumarium* treatment (SP for JNK inhibition, SB for p38 MAPK inhibition, PD for ERK inhibition, LY for PI3K/Akt inhibition).

### 2.12. In Vivo FeCl_3_-Induced Thrombus Model

Four groups of ICR mice (*n* = 5) were orally administered saline (negative control), ASA (100 mg/kg) (positive control), or *X. strumarium* (100 and 200 mg/mL) for 7 days to assess the development of FeCl_3_-induced thrombus formation. The FeCl_3_-induced thrombus model was established in ICR mice based on a study by Shim et al. (2021), which demonstrated that in terms of dose responses related to thrombus development and stability, ICR mice exhibit superior performance compared to C57BL/6 N mice [41,42]. Briefly, 1 h after the last dose of treatment, ICR mice were anesthetized via intraperitoneal injection of freshly prepared Avertin. After separating fat, fascia, and nerves, the left carotid was located, and blood flow was measured in the carotid artery of mice in the various study groups.

### 2.13. Drug-Likeness Analysis

SwissADME, a free web tool, was used to evaluate the pharmacokinetics and drug-likeness as reported previously [43,44]. Briefly, the canonical SMILES for the compounds were retrieved from PubChem (http://pubchem.ncbi.nlm.nih.gov/ (accessed on 26 November 2025)), and these SMILES strings were then entered into SwissADME (http://www.swissadme.ch/ (accessed on 26 November 2025)). The resulting output files and images were directly imported from the website. The BOILED-Egg (Brain Or Intestinal Estimated permeation predictive) model offers a fast and straightforward method to assess human intestinal absorption (HIA) and blood–brain barrier (BBB) permeation by calculating the lipophilicity and polarity of the molecules, subsequently generating a WLOGP versus tPSA plot.

### 2.14. Statistical Analysis

The collected data underwent a one-way analysis of variance (ANOVA), followed by a post hoc Dunnett’s test (GraphPad Prism 8.4.3) to assess the statistical significance of the observed variations. Results are expressed as mean ± standard deviation (SD). Statistical significance was established at a *p*-value of 0.05 or lower.

## 3. Results

### 3.1. X. strumarium Inhibits Agonist-Induced Platelet Aggregation

**Figure 1:**
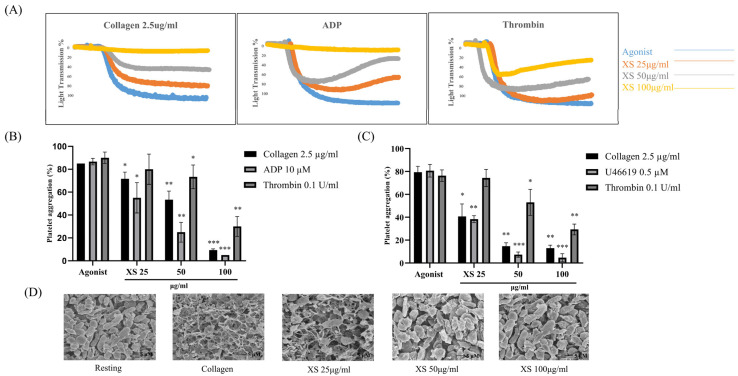
***X. strumarium* inhibits agonist-induced platelet aggregation, platelet shape changes.** Washed platelets were incubated for 1 min with *X. strumarium* (25, 50, and 100 µg/mL) in the presence of 1 mM CaCl_2_, and the platelet aggregation was then induced after adding agonists. (**A**) Representative light transmission aggregometry showing the effect of *X. strumarium* on collagen (2.5 µg/mL), ADP (10 µM), and thrombin (0.1 U/mL)-induced platelet aggregation in washed rat platelets. (**B**) Quantification of maximal platelet aggregation (%) following stimulation with collagen, ADP, or thrombin in the presence or absence of *X. strumarium*. (**C**) Inhibitory effects of *X. strumarium* on collagen (2.5 µg/mL), thrombin (0.1 U/mL), and U46619 (0.5 µM)-induced platelet aggregation in washed human platelets. (**D**) Scanning electron microscopy (SEM) images showing platelet morphology in resting, collagen-stimulation, and after *X. strumarium* pretreatment. *X. strumarium* markedly preserved resting morphology and inhibited activation-induced shape changes. Scale bars = 5 µm. Scale bars = 5 µm. Quantification of maximal aggregation expressed as percent of vehicle control (mean ± SD, *n* = 3). Data were analyzed by one-way ANOVA with Dunnett’s post hoc test. * *p* < 0.05, ** *p* < 0.01, and *** *p* < 0.001 compared with the agonist-treated group.

The antiplatelet effects of *X. strumarium* were assessed with light transmission aggregometry. Our results demonstrated that *X. strumarium* exhibited potent antiplatelet effects at 25, 50, and 100 μg/mL when rat and human platelets were stimulated in the presence of different agonists, including collagen at 2.5 μg/mL, ADP at 10 μM, U46619 at 0.5 μM, or thrombin at 0.1 U/mL. The platelet aggregation was substantially and concentration-dependently inhibited at 25, 50, and 100 µg/mL (*p* < 0.05 at 25, *p* < 0.01 at 50, and *p* < 0.001 at 100 µg/mL) (Figure 1A,B for rat platelets and 1C for human platelets). Furthermore, agonist-induced platelet shape changes were evaluated using scanning electron microscopy (SEM). SEM images showed that collagen causes platelets to change from a discoid to a rounded shape with filopodia, a transformation that was prevented by *X. strumarium* (Figure 1D). Scale bars = 5 µm. Moreover, no cytotoxic effects were observed at the indicated concentrations (Supplementary Figure S1). These findings are consistent with our previously published MTT assay, which demonstrated that *X. strumarium* extract at concentrations of 50, 100, and 200 µg/mL exhibited no cytotoxicity toward MH-S macrophage cells and exerted anti-inflammatory activity [31].

### 3.2. X. strumarium Inhibits ATP Release and [Ca^2+^]_i_ Mobilization

**Figure 2:**
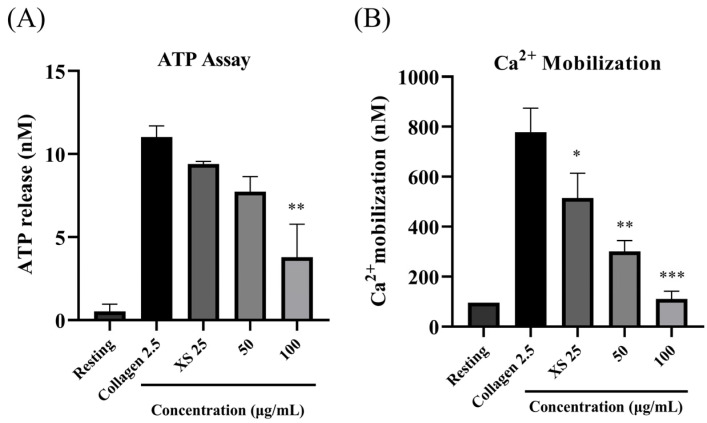
***X. strumarium* inhibits platelet granular release.** Platelets were preincubated with the *X. strumarium* and then stimulated with collagen to induce platelet activation. (**A**) The supernatant was used for the ATP assay using standard ATP assay kits. Data are presented as a percentage of vehicle control (mean ± SD, *n* = 3). * *p* < 0.05, ** *p* < 0.01 compared with collagen (one-way ANOVA with Dunnett’s post hoc test). (**B**) Additionally, [Ca^2+^]_i_ levels were analyzed using Fura-2/AM-loaded platelets. Data represent peak fluorescence ratio (F340/F380) normalized to vehicle (mean ± SD, *n* = 3); significance as in (**A**). * *p* < 0.05, ** *p* < 0.01, and *** *p* < 0.001 versus agonists.

ATP release and calcium mobilization assays were performed to evaluate dense granule secretion and platelet activation events, respectively. Calcium signaling, tightly regulated by ATP-dependent SERCA pumps, is essential for the intracellular Ca^2+^ rise that triggers platelet activation and aggregation [45]. Our results revealed a dose-dependent inhibition of ATP and calcium mobilization release at 25, 50, and 100 μg/mL (Figure 2A,B). These findings demonstrate that *X. strumarium* not only impairs GPVI-mediated platelet aggregation (Figure 1) but also suppresses key downstream events such as dense-granule secretion and calcium signaling in a concentration-dependent manner (Figure 2B).

### 3.3. X. strumarium Downregulates Inside-Out and Outside-In Signaling

**Figure 3:**
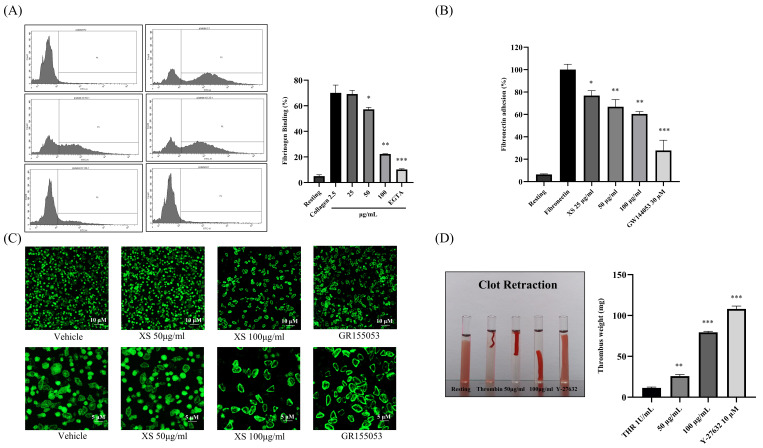
***X. strumarium* inhibits integrin αIIbβ3-mediated inside-out and outside-in signaling.** (**A**) Flow cytometric analysis of fibrinogen binding to integrin αIIbβ3 was performed to assess inside-out signaling. Washed rat platelets were preincubated with *X. strumarium* (25, 50, or 100 µg/mL), followed by stimulation with collagen (2.5 µg/mL), and stained with anti-fibrinogen-FITC antibody. The percentage of fibrinogen-positive platelets was quantified. (**B**) Washed rat platelets were pre-incubated with different concentrations of *X. strumarium*, vehicle, or GR144053 and then loaded onto a fibronectin-coated plate and incubated for 90 min at 37 °C. Then, the assay was performed. (**C**) Platelets were pretreated with vehicle, *X. strumarium* (50 or 100 µg/mL), or GR155053 and allowed to adhere to fibrinogen-coated coverslips for 60 min at 37 °C. Cells were fixed and stained with phalloidin, and then imaged using fluorescence microscopy. Scale bars: 5–10 µm. (**D**) The clot retraction assay was used to evaluate outside-in signaling. Human platelet-rich plasma (PRP) was pretreated with *X. strumarium* (25, 50, or 100 µg/mL) or Y-27632 (negative control), followed by stimulation with human thrombin (1 U/mL). Clot retraction was visually monitored at room temperature. Data are expressed as mean ± SD (*n* = 3) for the percentage of fibrinogen-positive cells or clot retraction. Statistical analysis was performed using one-way ANOVA followed by Dunnett’s post hoc test. * *p* < 0.05, ** *p* < 0.01, *** *p* < 0.001 vs. agonist- or thrombin-treated group.

Upon activation, platelet integrin αIIbβ3 undergoes a conformational change that increases its affinity for bivalently bound fibrinogen—a process known as inside-out signaling. The binding of fibrinogen to integrin αIIbβ3 then initiates outside-in signaling, which reinforces platelet activation, cytoskeletal rearrangement, and clot retraction. To evaluate the effects of *X. strumarium* on these processes, we assessed its impact on collagen-induced platelet signaling. Our results showed that *X. strumarium* significantly inhibited fibrinogen binding to integrin αIIbβ3 (Figure 3A), suggesting suppression of inside-out signaling. In addition, the impact of *X. strumarium* on platelet fibronectin adhesion (Figure 3B) and cytoskeletal spreading was evaluated on fibrinogen-coated coverslips using phalloidin staining to detect F-actin organization (Figure 3C). Vehicle-treated platelets exhibited extensive spreading characterized by large, flattened morphology and well-developed actin-rich lamellipodia, indicating robust integrin αIIbβ3-mediated outside-in signaling. Pretreatment with *X. strumarium* resulted in a concentration-dependent reduction in platelet spreading. At 50 µg/mL, platelets adhered to the fibrinogen surface but displayed smaller contact areas with fewer lamellipodial extensions, consistent with partial inhibition of actin polymerization. The inhibitory effect was more pronounced at 100 µg/mL, where the majority of platelets remained rounded or minimally spread, showing marked suppression of cytoskeletal rearrangement. GR155053, a selective αIIbβ3 antagonist, almost completely abolished platelet spreading, producing predominantly rounded platelets with minimal actin extension (Figure 3C). This positive control confirms that the morphological changes observed with *X. strumarium* are mediated by impaired integrin-dependent outside-in signaling. Additionally, the extract reduced clot retraction, indicating an inhibitory effect on outside-in signaling and platelet shape change (Figure 3D).

### 3.4. X. strumarium Attenuates MAPK and PI3K/Akt Phosphorylation

**Figure 4:**
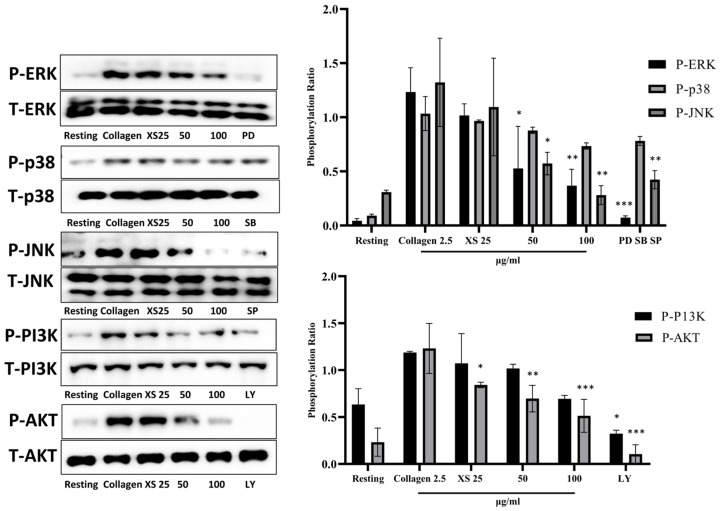
**MAPK and PI3K/Akt phosphorylation were inhibited after treatment with *X. strumarium*.** Washed rat platelets were preincubated with XS (25, 50, or 100 µg/mL) for 1 min, followed by stimulation with collagen (2.5 µg/mL) for 5 min. Reactions were terminated by adding ice-cold lysis buffer. Protein concentrations were determined, and equal amounts of protein were separated by SDS-PAGE and transferred onto PVDF membranes. After blocking, membranes were probed with antibodies specific for total and phosphorylated forms of ERK, p38, JNK, PI3K, and Akt. Protein bands were visualized using enhanced chemiluminescence (ECL). Pathway inhibitors as negative controls: PD–ERK inhibitor, SB–p38 MAPK inhibitor, SP–JNK inhibitor, LY–PI3K/Akt pathway inhibitor. Quantification of the ratio of phosphorylated to total protein, normalized to vehicle control (mean ± SD, *n* = 3). * *p* < 0.05, ** *p* < 0.01, and *** *p* < 0.001 compared with collagen.

Phosphorylation within the MAPK and PI3K/Akt pathways plays a critical role in platelet activation. The upregulation of MAPK facilitates integrin activation, granule secretion, and cytoskeletal rearrangements, which are essential for the initial steps of platelet adhesion and aggregation. Concurrently, PI3K/Akt phosphorylation contributes to the stabilization of platelet aggregates by promoting actin polymerization and further enhancing integrin signaling. Western blot results demonstrated significant inhibition of the phosphorylation of MAPK and PI3K/Akt with *X. strumarium* (Figure 4).

### 3.5. X. strumarium Prevents Thrombosis and Regulates Hemostasis

**Figure 5:**
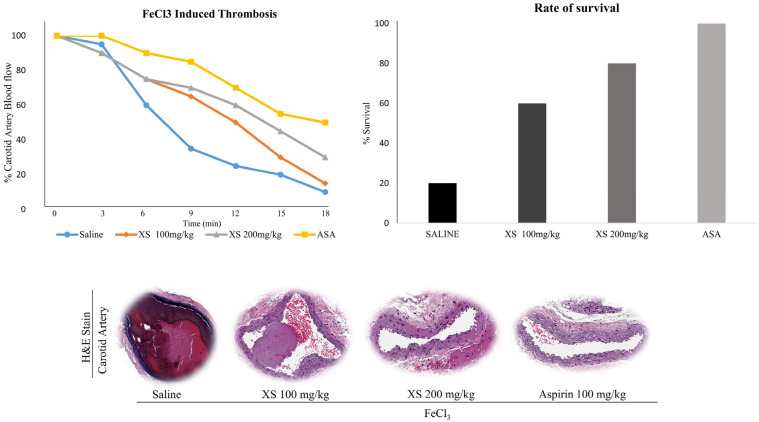
***X. strumarium* prevents in vivo FeCl_3_-induced thrombosis.** The FeCl_3_-induced thrombus model was established in ICR mice (*n* = 5) (Group 1 received saline, Group 2 received ASA (100 mg/kg), and Groups 3 and 4 received doses of *X. strumarium* (100 and 200 mg/mL). Data represent the means ± standard error of the mean.

The FeCl_3_-induced thrombosis model is a well-established and widely accepted model for evaluating acute arterial thrombosis, platelet vessel wall interaction, and the efficacy of antithrombotic agents [20,41,46]. It allows for quantifiable, reproducible, and rapid assessment of thrombus formation in vivo, particularly in response to vascular injury, a critical early event in thrombosis [46]. Following thrombus induction using 35% FeCl_3_, treatment with *X. strumarium* resulted in improved blood flow and increased survival rates of mice, comparable to those observed in the saline group (no treatment group) and aspirin (ASA) as a positive control (Figure 5).

### 3.6. Identification of the Bioactive Compounds, Pharmacokinetics, and Drug-likeness

**Figure 6:**
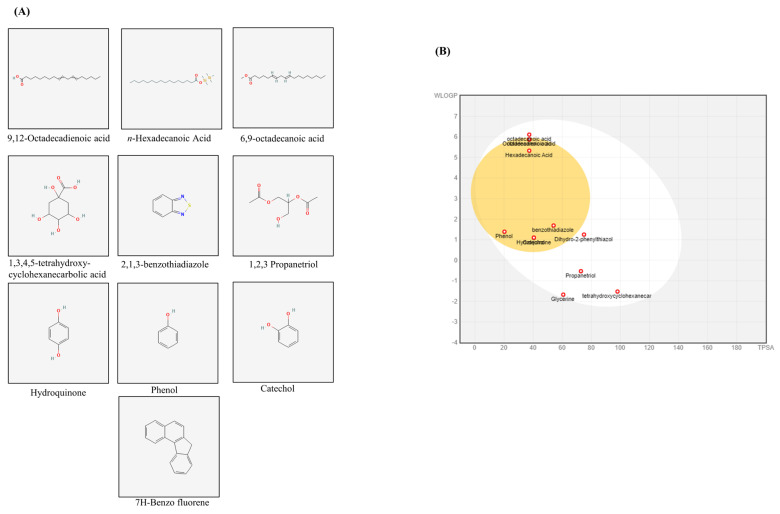
**BOILED-Egg analysis and bioavailability of active compounds.** Selective compounds were sourced from PubChem in the form of a two-dimensional (2D) SDF file (**A**). The canonical SMILES for the obtained compound were retrieved from PubChem, and these SMILES strings were then entered into SwissADME. The resulting output files and images were directly imported from the website. The BOILED-Egg assessed human intestinal absorption (HIA) and blood–brain barrier (BBB) permeation by calculating the lipophilicity and polarity of the molecules, subsequently generating a WLOGP versus tPSA plot (**B**).

The GC-MS analysis of *X. strumarium* revealed several major compounds, including 9,12-octadecadienoic acid, *n*-hexadecanoic acid, 6,9-octadecanoic acid, octadecanoic acid, 1,3,4,5-tetrahydroxycyclohexanecarboxylic acid, 2,1,3-benzothiadiazole, 1,2,3-propanetriol, glycerin, hydroquinone, catechol, phenol, 5,6-dihydro-2-phenylthiazol, and 2,4-dimethyl-7H-benzofluorene (Supplementary Figure S2 and Table S1). Standard peaks were obtained, and two-dimensional (2D) chemical structures of major compounds acquired from PubChem (NHI) are presented in Figure 6A. Further, SwissADME was used to calculate bioavailability scores and drug-likeness as reported previously [47] (Figure 6B). We used OB ≥ 30 and drug-likeness ≥ 0.18 to evaluate the chemical compounds from *X. strumarium*. When comparing these compounds, catechol, 2,1,3-benzothiadiazole, and hydroquinone were found to possess superior pharmacological properties.

## 4. Discussion

Collagen, thrombin, U46619, and ADP activate GPVI, PAR, TXA2, and P2Y12 receptors, leading to strong platelet aggregation. The most pronounced reduction in rats and human platelets after treatment with *X. strumarium* was induced by these agonists (collagen, ADP, U46619, and thrombin). *X. strumarium* at 100 μg/mL completely abolished these agonist-induced platelet activation and aggregation. Furthermore, platelet shape changes were assessed using SEM. SEM images revealed a morphological shift in platelets from a discoid to a rounded form, characterized by the presence of filopodia upon collagen activation. This shape change was effectively prevented, maintaining the original discoid structure, when treated with *X. strumarium* [39].

Platelet α-granules are rich in adhesive ligands such as fibrinogen and fibronectin, as well as membrane proteins like P-selectin, whereas dense (δ)-granules primarily store small molecules including Ca^2+^, ADP, and ATP. Platelet activation leads to the release of these granules, fostering platelet adhesion, morphological alterations, and aggregation [48]. *X. strumarium* was observed to inhibit α- and δ-granule secretion, thus reducing platelet activation, adhesion, shape change, and aggregation, corroborating previous findings by Holmsen [49]. The final step of platelet aggregation involves inside-out signaling and integrin αIIbβ3 activation, which is crucial for this process [50]. Upon activation, integrins on adjacent platelets exhibit high-affinity binding to bivalently bound fibrinogen molecules, facilitating the formation of stable platelet aggregates through αIIbβ3-fibrinogen bridges. Under high-shear flow conditions, integrin-dependent interactions with VWF also contribute to aggregate formation [51] while low-level signaling through the GPIb–V–IX complex can facilitate the binding of fibrinogen to αIIbβ3, promoting platelet aggregation [52]. In our experiment setup, when αIIbβ3 activation was assessed after incubating platelets with fibrinogen-binding antibodies, FACS analysis results showed that *X. strumarium* significantly inhibited αIIbβ3 activation and inside-out signaling at 100 μg/mL. Rho kinases, which are downstream regulators that phosphorylate the myosin light chain in response to RhoA, play a pivotal role in clot retraction and were also significantly inhibited [53]. Additionally, MAPK and PI3K/Akt are critical in platelet activation, influencing calcium mobilization, granule secretion, and aggregation [20,44,50]. MAPKs, including ERK, p38 MAPK, and JNK, have been extensively studied to be involved in collagen-induced platelet activation [54], while phosphoinositide 3-kinase (PI3K) plays an important role in GPVI-mediated platelet activation [55]. Akt activation is regulated by the level of phosphoinositide 3-phosphates (PI3K) [56], and platelets with a lack of Akt have defects in the secretion of dense and α-granule contents [57]. In addition, collagen- and thrombin-induced ATP secretion are reduced in the absence of Akt or in the presence of the Akt inhibitor [58]. In this study, *X. strumarium* effectively inhibits these pathways, resulting in reduced MAPK and PI3K/Akt phosphorylation, offering mechanistic inhibition of platelet activation and aggregation. Furthermore, the inhibitory effects of *X. strumarium* on hemostasis were assessed using a FeCl_3_-induced thrombus model. Treatment with *X. strumarium* significantly reduced thrombus formation and modestly improved blood flow and animal survival compared to treatment with ASA positive control. Additional validation in models mimicking atherosclerosis-related thrombosis (e.g., ApoE^−^/^−^ mice or high-fat diet-induced models) would further enhance the translational relevance of this study.

Our findings suggest that *X. strumarium* possesses potent chemical constituents that lead to antiplatelet activities, as revealed by GC-MS analysis. The presence of these active compounds (9,12-octadecadienoic acid, *n*-hexadecanoic acid, 6,9-octadecanoic acid, 1,2,3-propanetriol, hydroquinone, catechol, and phenol) in *X. strumarium* extracts likely contributes to these beneficial pharmacological effects, as these components have been previously reported for their therapeutic properties [59,60,61,62,63,64,65,66,67,68]. Wenxiang Fan et al. reported traditional and clinical uses of *X. strumarium* in tablet and pill forms [27]. Previously, catechol (0.5–5 µmol/animal; 1–200 µM) and hydroquinone (50–5000 nmol/mouse; 1–250 µM) were shown to exhibit in vivo antiplatelet effects without toxicity [69,70]. A recent in vivo study assessing the potential toxicity of *X. strumarium* seeds in rats demonstrated that even a high dose of cocklebur seed extract caused no histopathological, hematological, or biochemical abnormalities, indicating safety in the rat model [71]. Moreover, Amin et al. [34] reported the pharmacokinetic characteristics of several major compounds in *X. strumarium* using in silico ADMET models (SwissADME and pkCSM). Key constituents demonstrated favorable absorption (moderate water solubility), distribution (acceptable VDss and BBB permeability), metabolism (non-inhibitory toward major CYP enzymes), and excretion (moderate clearance values). Predicted LD50 values suggest a low acute toxicity risk, and most compounds were predicted to be non-hepatotoxic. SwissADME was applied to evaluate compounds from *X. strumarium,* and our results showed that catechol, 2,1,3-benzothiadiazole, and hydroquinone had the highest predicted GI absorption and blood–brain barrier permeability. Additionally, these compounds met key drug-likeness criteria by not violating the Lipinski, Veber, and Egan filters. Literature review revealed that catechol inhibits platelet aggregation in vitro (1–200 µM) and ex vivo (0.5–5 µmol/animal) via ERK/p38 pathways [69], and Hydroquinone reduces AA-induced aggregation (1–250 µM) without cytotoxicity [70], while 2,1,3-Benzothiadiazole appears to be a novel candidate with no prior platelet data. Finally, a network pharmacology approach was employed to explore the mechanistic basis of the antiplatelet effects of these pharmacologically superior components (Supplementary Figure S3). The Venn diagram revealed 26 overlapping genes between the predicted targets of the identified compounds and known platelet-related genes. A STRING protein–protein interaction (PPI) network illustrated strong interactions among key nodes, including NFKB1, STAT1, MTOR, CXCR4, and SLC2A1, indicating a tightly interconnected regulatory network (Supplementary Figure S3). KEGG pathway mapping identified several inflammation and metabolism-related pathways enriched by these targets, such as toll-like receptor signaling, HIF-1 signaling, adipocytokine signaling, regulation of actin cytoskeleton, and chemokine signaling. These findings suggest that the antiplatelet effect of *X. strumarium* may be mediated through indirect modulation of immune-metabolic and cytoskeletal pathways.

While the current findings provide important mechanistic insight, future studies investigating the neurotoxicity and individual or combined effects of these compounds using appropriate in vitro and in vivo models will be essential to determine their individual pharmacological activities, therapeutic window, clinical suitability, define safe dosage ranges, and minimize the risk of unintended systemic or CNS-related effects.

## Data Availability

The original contributions presented in the study are included in the article/Supplementary Material. Further inquiries can be directed to the corresponding authors.

## Supplementary Materials

The following supporting information can be downloaded at: https://www.mdpi.com/article/10.3390/biomedicines13122924/s1, Supplementary Figure S1. LDH release assay, Figure S2. GC-MS analysis peaks, Figure S3. network pharmacological analysis, and Figure S4 the full-length blots for the gel images, Table S1 major chemical compounds.

## Abbreviations

The following abbreviations are used in this manuscript:

Abbreviation	Full Form/Description
ACD	Acid-citrate-dextrose
ADP	Adenosine diphosphate
Akt	Protein kinase B
[Ca^2+^]_i_	Intracellular calcium concentration
CVD	Cardiovascular disease
ERK	Extracellular signal–regulated kinase
FeCl_3_	Ferric chloride
ICR	Institute of Cancer Research (mouse strain)
IRB	Institutional Review Board
JNK	c-Jun N-terminal kinase
KEGG	Kyoto Encyclopedia of Genes and Genomes
LDH	Lactate dehydrogenase
MAPK	Mitogen-activated protein kinase
OB	Oral bioavailability
PAR	Protease-activated receptor
PFA	Paraformaldehyde
PI3K	Phosphoinositide 3-kinase
PPi	Protein–protein interaction
PRP	Platelet-rich plasma
PVDF	Poly (vinylidene fluoride)
RhoA	Ras homolog family member A
ROCK	Rho-associated coiled-coil containing protein kinase
SEM	Scanning electron microscopy
STAT1	Signal transducer and activator of transcription 1
TXA_2_	Thromboxane A_2_
tPSA	Topological polar surface area
VWF	von Willebrand factor
WLOGP	Wildman–Crippen LogP (lipophilicity descriptor)
*X. strumarium*	*Xanthium strumarium*
