# Supplementary material for: Xanthium strumarium L. Exhibits Potent Antiplatelet and Antithrombotic Effects by Modulating MAPK and PI3K/AKT Signaling Pathways and Inhibiting Ferric Chloride-Induced Thrombosis
Source: Biomedicines. 2025 Nov 28;13(12):2924. doi: 10.3390/biomedicines13122924 (PMC12731137; doi:10.3390/biomedicines13122924)
Supplement: Supplementary file 1 [file biomedicines-13-02924-s001.zip › biomedicines-3966602-supplementary.pdf]

Xanthium strumarium L. exhibits potent antiplatelet and antithrombotic effects by modulating MAPK and PI3K/AKT signaling pathways and inhibiting ferric chloride-induced thrombosis

Abdul Wahab Akram [1], Ga Hee Lee[2], Su-Min Baek[1], Jinsu Kang[1], Yoonhoi Koo[1], Yein Oh[1], Min-Soo Seo[1], Evelyn Saba[3], Dong-Ha Lee[2], Man Hee Rhee[1,4] †

[1] Department of Veterinary Medicine, College of Veterinary Medicine, Kyungpook National University, Daegu 41566, Republic of Korea

[2] Department of Biomedical Laboratory Science, Molecular Diagnostics Research Institute, Namseoul University

[3] Department of Veterinary Biomedical Sciences, Faculty of Veterinary and Animal Sciences, Pir-Mehr Ali Shah Arid Agriculture University, Rawalpindi 46000, Pakistan

[4] Institute for Veterinary Biomedical Science, College of Veterinary Medicine, Kyungpook National University, Daegu 41566, Republic of Korea

†Corresponding author: Man Hee Rhee, PhD

Laboratory of Physiology and Cell Signaling, College of Veterinary Medicine, Kyungpook National University, Daegu 41566, Republic of Korea
Tel: +82-53-950-5967, 010-6753-2531; Fax: +82-53-950-5955

Email address: rheemh@knu.ac.kr

Supplementary Figure S1

To assess the cytotoxicity of *X. strumarium* extract on platelets, an LDH release assay was conducted after 1 hour of incubation at concentrations of 25, 50, 100, and 200 µg/mL. As shown in Figure S1, treatment with *X. strumarium* did not induce any significant increase in LDH release compared to the collagen control (2.5 µg/mL), indicating that the extract did not compromise platelet membrane integrity at any tested concentration. Cell viability remained above 95% across all groups, confirming the extract's non-cytotoxic profile under the experimental conditions.

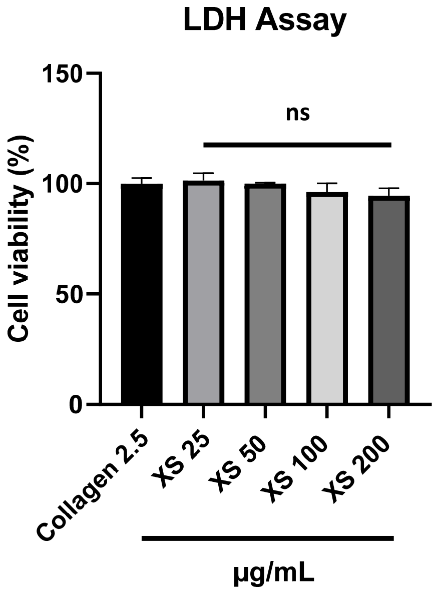

Supplementary Figure S1. *X. strumarium* (XS) extract does not induce cytotoxicity in platelets. Washed platelets (2.5 × 10^8/mL) were incubated with varying concentrations of XS (25–200 μg/mL) for 1 hour. LDH release in the supernatant was measured using an ELISA reader at 450 nm. Data are expressed as mean ± SEM (n = 3). No significant difference (ns) was observed compared to the collagen control group (2.5 μg/mL), indicating high cell viability.

Supplementary Figure S2

GC-MS analysis and major chemical compounds identified in *X. strumarium* extract. The standard peaks obtained are shown.

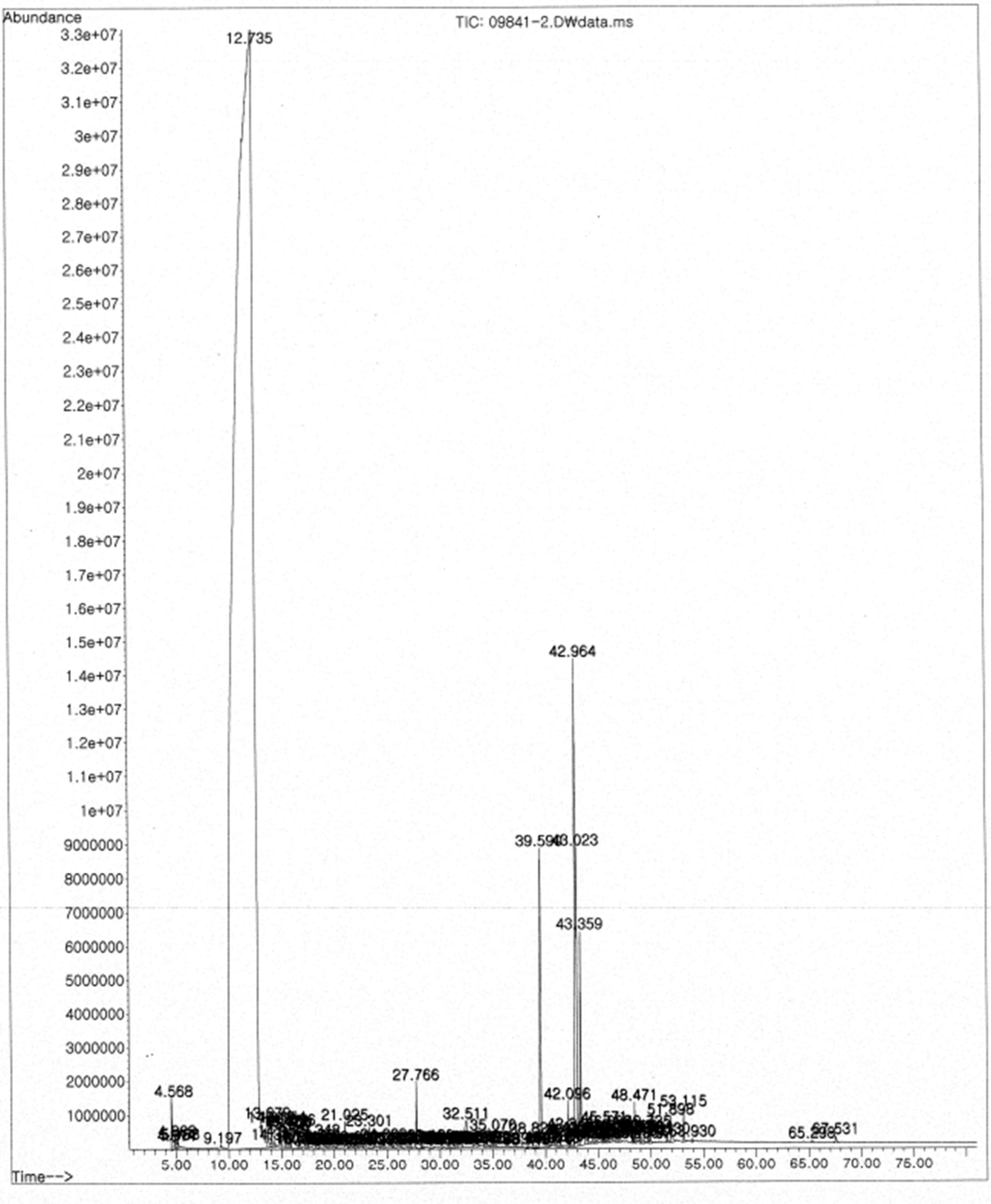

Supplementary Figure S3

To investigate the mechanistic basis of the antiplatelet effects of individual compounds from *X. strumarium*, a comprehensive network pharmacology analysis was conducted (Fig. S3A-C). As a compound target in Fig. S3D, the Venn diagram identified 26 overlapping target genes between the predicted targets of catechol, hydroquinone, and 2,1,3-benzothiadiazole and platelet-associated genes, suggesting a potential molecular link between these compounds and platelet-regulatory pathways.

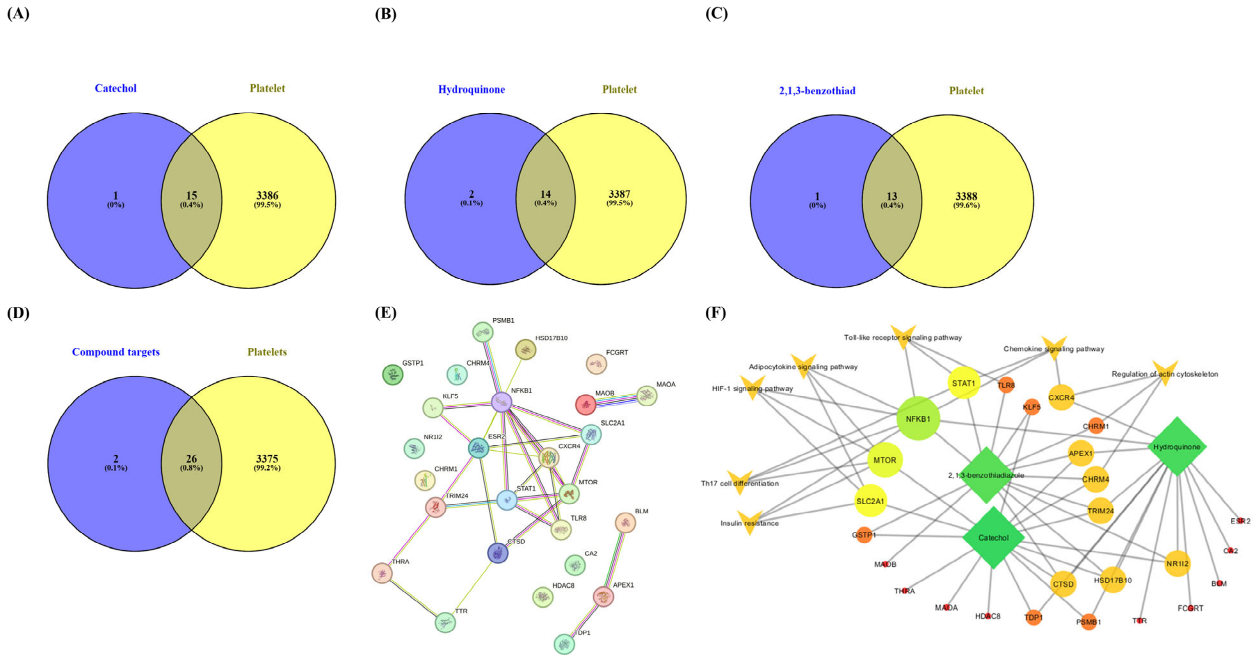

Supplementary Figure S3. Network pharmacology analysis of *X. strumarium* compounds related to platelet function. Venn diagram showing 26 overlapping target genes between predicted compound targets (catechol, hydroquinone, 2,1,3-benzothiadiazole) and platelet-associated genes. Protein–protein interaction (PPI) network of overlapping genes generated using the STRING database (Homo sapiens). Compound–target pathway interaction network generated using Cytoscape. Diamonds represent compounds; circles represent target genes; triangles represent enriched KEGG pathways. Node size and color reflect network degree and pathway relevance.

Supplementary Figure S4

After preincubating with *X. strumarium* and stimulation with collagen, lysis buffer was added to stop the reaction and start lysis. The resulting protein concentration was measured, and whole platelet proteins were isolated for subsequent analysis. These proteins were separated using sodium dodecyl sulfate-polyacrylamide gel electrophoreses (SDS-PAGE), transferred to poly(vinylidene fluoride) (PVDF) membranes, membranes were then blocked, and primary antibodies were applied overnight. The membranes were then treated with secondary antibodies for 3 h and washed three times for visualization using enhanced chemiluminescence. This comprehensive process allowed for the examination of changes in protein expression induced by *X. strumarium* treatment.

Western blot results demonstrated significant inhibition of the phosphorylation of MAPK and PI3K/Akt with different doses of *X. strumarium*. The full-length blots for the gel images can be presented as supplementary figure S3 = P-ERK pathway; P-p38 pathway; P-JNK pathway; P-Akt pathway; PI3K pathway in platelets.

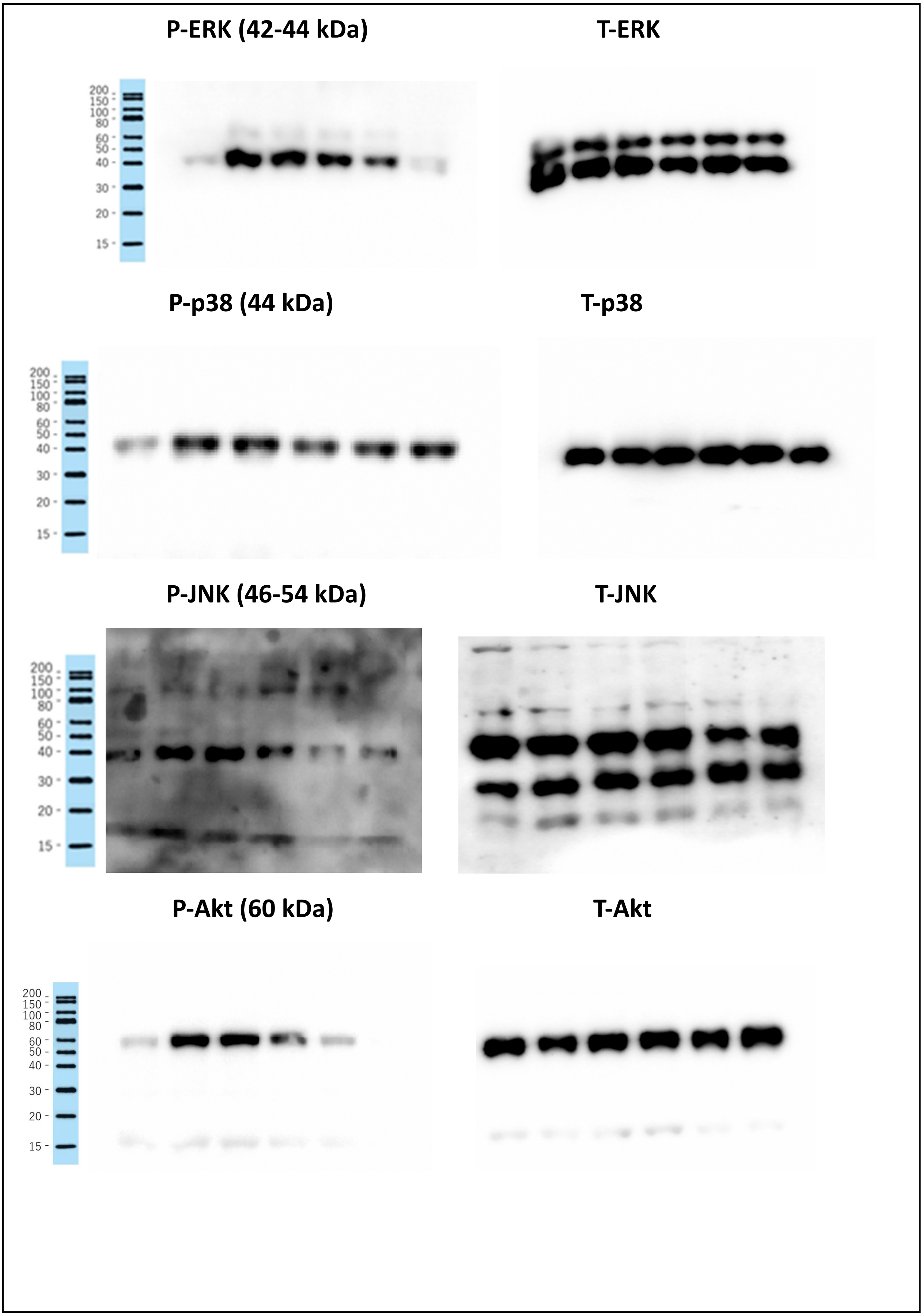
P-ERK (42-44 kDa)
T-ERK
P-p38 (44 kDa)
T-p38
P-JNK (46-54 kDa)
T-JNK
P-Akt (60 kDa)
T-Akt
200
150
100
80
60
50
40
30
20
15

P-PI3K (85 kDa)

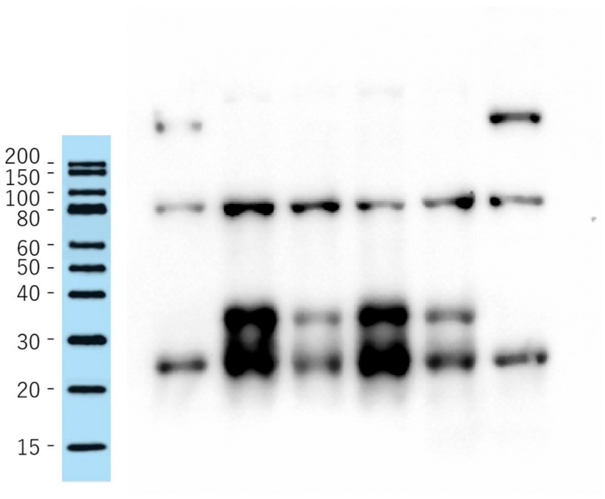

T-PI3K

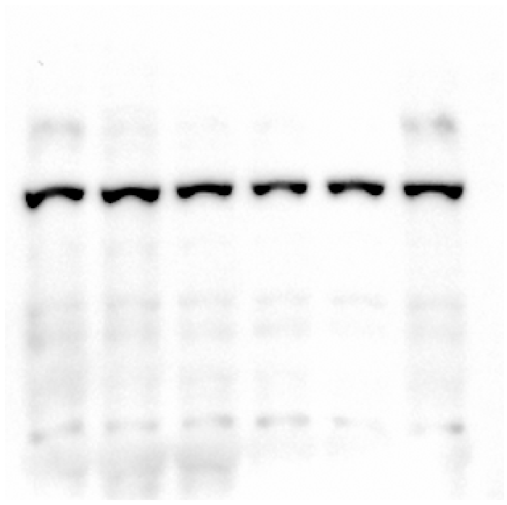

Supplementary Table S1. The GC-MS analysis revealed several major compounds.

S.no.	RT	Area %	Chemical compound	Molecular weight g/mol	Molecular formula
1	42.96	2.94	9,12-Octadecadienoic acid	280.4	$C_{18}H_{32}O_2$
2	39.59	1.35	*n*-Hexadecanoic Acid	386.8	$C_{21}H_{46}O_2Si_2$
3	43.02	0.73	6,9-octadecanoic acid	294.5	$C_{19}H_{34}O_2$
4	43.35	0.63	octadecanoic acid	282.5	$C_{18}H_{34}O_2$
5	32.51	0.39	1,3,4,5-Tetrahydroxycyclohexanecarboxylic acid	192.17	$C_7H_{12}O_6$
6	27.76	0.23	2,1,3-benzothiadiazole	136.18	$C_6H_4N_2S$
7	16.02	0.22	1,2,3 Propanetriol	190.19	$C_8H_{14}O_5$
8	15.42	0.11	Glycerin	92.09	$C_3H_8O_3$
9	23.30	0.09	Hydroquinone	110.11	$C_6H_4(OH)_2$
10	21.02	0.08	Catechol	110.11	$C_6H_6O_2$
11	15.15	0.09	Phenol	94.11	C_6H_6O
12	48.47	0.07	5,6-Dihydro-2-phenylthiazol	163.24	C_9H_9NS
13	53.11	0.07	2,4-Dimethyl-7H-benzo fluorene	244.3	$C_{19}H_{16}$
